# The determinants of the propensity to receive publicly funded home care services for the elderly in Canada: a panel two-stage residual inclusion approach

**DOI:** 10.1186/s13561-016-0086-6

**Published:** 2016-02-25

**Authors:** Gustavo Mery, Walter P. Wodchis, Audrey Laporte

**Affiliations:** 1 Institute of Health Policy, Management and Evaluation, University of Toronto, Health Sciences Building, 155 College Street, Suite 425, Toronto, ON M5T3M6 Canada; 2 Canadian Centre for Health Economics/Centre canadien en économie de la santé, 155 College Street, Toronto, ON M5T3M6 Canada

**Keywords:** Home care, Elderly, Long-term care, Public provision, Complementary effect, Determinants

## Abstract

The role of Home Care (HC) services for the elderly will be increasingly important in meeting populations’ future needs for care. HC services include Home Health Care (HHC) and Homemaking/Personal Support (HMPS), distinction rarely seen in the literature. This paper argues that it is important to distinguish between these types of HC, since the factors that drive the likelihood of the receipt of each type of care may differ, and also to investigate the interrelationship between them. We explored the interrelationship between receipt of publicly funded HMPS and HHC, and the determinants of the receipt of each type of services. A Panel Two-Stage Residual Inclusion approach was applied to estimate the likelihood of the receipt of HC services using data for those aged 65 and over from 9 biannual waves of the Canadian National Population Health Survey (1994-95 to 2010-11). We found that there are in fact differences in the determinants of the likelihood of HHC and HMPS receipt. Moreover, receipt of publicly funded HMPS was found to be complementary with receipt of publicly funded HHC services after adjusting for functional and health status. Dependence on help with activities of daily living, health status, household arrangement, and income were found to be determinants of the propensity to receive both publicly funded HHC and HMPS services. This study aims to contribute to the existent literature by taking a step toward explicitly modelling the potential interaction between the determinants of the receipt of different types of HC services simultaneously, as a system. Our methodological approach, a Panel Two-Stage Residual Inclusion method, seems to effectively address problems that are known to be a source of bias in the literature.

## Background

One anticipated consequence of the aging of societies around the world is an increase in the prevalence of chronic conditions and disability and a higher demand for long-term care, including home care (HC) services. The need for long-term care services is expected to dramatically increase worldwide, at least during the coming four decades [1, 2]. By 2050, the number of people around the world 80 years of age and older will increase fourfold, and the global dependency ratio for this age group, relative to the ratio for the population aged 15 to 64, will increase more than threefold [2]. The provision of social care services is likely to grow much faster than the provision of medical treatments by hospitals and doctors [3, 4]. Consequently, the role of HC services will be increasingly important in meeting populations’ future needs for care.

In this study, the distinction between Home Health Care (HHC) services and Homemaking/Personal Support (HMPS) services is made. HHC services include nursing care, physiotherapy, nutritional counselling, and other health care services delivered by professional health care staff. In contrast, HMPS services help people with daily tasks, such as meal preparation, eating, toileting, personal hygiene, medication reminders, laundry, light housekeeping, shopping, and transportation [5–7]. These are usually referred to as Activities of Daily Living/Instrumental-ADLs (ADL/IADLs). Most HHC services are provided by paid (formal) caregivers (e.g., community nurses), while HMPS services are provided primarily by either paid (e.g., personal support workers) or informal, unpaid caregivers (e.g., spouses, family members, or friends).

Despite the differences between HHC and HMPS services, distinctions are often not made in the literature. The distinction may be an important one however, since there are likely to be differences in the determinants of the demand for these two types of services. For example, an early discharge from hospital may be supported by post-acute nursing care at home, while a senior suffering from dementia will mostly require help with ADL/IADL. Equally important is the different potential for substitution for informal care. While a functional spouse will provide support with personal care, he or she will hardly be able to replace the role of providers of more medically intensive services. The need, access, availability of subsidies, and the potential for substitution among public, private, and informal care within each service type are all expected to differ.

There is also the issue that once an individual has received either HHC or HMPS services that this may influence the likelihood that they receive the other type of service. Most jurisdictions in North America and Europe allocate HC services primarily based on users’ need. However, it might be the case, as with other health and social services, that once seniors enter the system, the likelihood of receipt of additional services is higher at lower levels of need. This represents not only an issue of inequitable allocation of scarce resources; with capped budgets and overall service volume constraints [8], receipt of HHC or HMPS services for factors other than need can translate into leaving those who are most deprived without any assistance.

The primary objectives of this study were to explore the determinants of the receipt of publicly funded HHC and HMPS; and by modelling the types of services jointly, to determine whether publicly funded HHC and HMPS services are complements or substitutes in the Canadian context. The analysis also pays particular attention to whether socio-economic factors affect the likelihood of the receipt of publicly funded HC services.

## Background literature

### The determinants of the receipt of HC services

Age and dependence on help with ADLs/IADLs have been consistently reported as factors that increase the receipt of HC services of any type and from any source [9–23] and, together with health status, are generally used to indicate users’ need for HC services. Household income and living arrangement are also among the determinants of HC receipt consistently reported in the literature, although their effect may be expected to vary considerably in different contexts. A summary of findings from the literature on the determinants of the receipt of HC services is presented in Table 1.

**Table 1:** Background literature – the determinants of the receipt of HC services

Determinant of HC receipt	Study reference	Country/region	Findings (or variable used if indicated)
Age	[9–23]	US, UK, The Netherlands, Sweden, Ontario, Finland, Europe.	Increase the receipt of HC services of any type and from any source. Together with health status, are generally used to indicate users’ need for HC services
Dependence on help with ADLs/IADLs			
Health status	[15, 16, 42]	US	Variables used: Self-rated health measure and a list of chronic conditions, including heart disease, stroke, diabetes, cancer, arthritis, and incontinence.
	[18]	The Netherlands	Variables used: Several chronic physical and mental conditions.
	[26]	Ontario	Variable used: Individual’s level of morbidity, characterized in terms of 12 clinical groupings.
Household income	[43]	The Netherlands	Higher utilization of publicly funded HC among lower income seniors, within a system that makes copayments proportional to income.
	[20]	Finland	Higher level of utilization of publicly funded HC services among higher income seniors, without copayments by users.
	[17]	Sweden	Did not find a significant effect of income on allocation of HMPS hours, within a system that also has no copayments.
	[15]	US	Nonsignificant differences in Medicare HC expenditures according to income, using merged data from the 1995 wave of the Asset and Health Dynamics Among the Oldest-Old (AHEAD) and the 1998 wave of the Health and Retirement Study.
	[16]	US	Lower Medicare HC expenditures for higher income seniors using the 1993 and 1995 waves of the AHEAD survey.
	[26]	Ontario	Higher receipt of and higher intensity of publicly funded HC services among adults who lived in low-income neighbourhoods.
Living arrangement	[9, 10, 20, 44, 45]	US, Finland, Canada	Higher levels of formal HC receipt among seniors who lived alone.
	[46]	Canada	Marital status has been found to be negatively associated with receipt of publicly funded HC.
	[42]	US	Marital status has been found to be negatively associated with any formal HC receipt.
	[17]	Sweden	Coresiding seniors were allocated significantly fewer hours of publicly funded HMPS than those who lived alone, adhering to the explicit allocation criteria for public services.
	[21]	Finland	Reported higher levels of publicly funded HMPS and HHC receipt among seniors who lived alone.

It is worth noting that all the studies cited in relation to the effect of income on HC made no distinction between HHC and HMPS services, with the exception of Meinow et al.’s [17], which only considered HMPS services. Consequently, we cannot infer from the literature whether the effect of income on receipt of publicly funded HHC and HMPS differs.

### The relationship between publicly funded HHC and HMPS services

U.S.-based studies generally do not make the distinction between HHC services and HMPS services, and they usually use the term “home health care” to refer to both health care and social services delivered at home. The main reason for this seems to be that Medicare and Medicaid fund both HHC and HMPS services for eligible users. In contrast, several European studies acknowledge this distinction [17, 21, 24]. This is not surprising given that in many countries in Europe, HHC services are part of the national health care system, while HMPS services fall under municipal governments.

Two recent European studies using 2004 data from the Survey of Health, Ageing and Retirement in Europe (SHARE), which includes data from 18 European countries, supports the importance of making the distinction between HHC and HMPS, since informal care was found to be a substitute for HMPS yet a complement to HHC [22] and to appointments with a doctor and hospital visits [25]. However, the interrelationship between publicly funded HHC and HMPS services was not directly explored.

In Canada, a number of studies have made the distinction between HHC and HMPS services, but only for descriptive purposes [19, 26–28]. Specific analysis of determinants of receipt of these two types of HC services has not been conducted.

Up to this point, the literature has treated HC as one homogenous type of services or has explored HHC and HMPS separately. However, in addition to the evident difference between these types of services, as we noted above, there is potentially a system of interactions between them that has not yet been explored. In order to effectively understand the determinants of the receipt of HC services, modeling the simultaneous effect of the receipt of different types of services is required. This study represents a step toward trying to explicitly model this interaction in an effort to address this research gap in the literature.

### The home care system in Canada

At the beginning of the 2000s, estimates suggested that approximately 20 % of formal HC services in Canada were privately financed, with the remaining 80 % financed by the public sector [26, 29]. The proportion of public HC funding in Canada devoted to HHC services rather than to HM grew from 43.3 % in 1994–1995 to 48.6 % in 2003–2004 [30]. Of all the care provided at home, an estimated 80 % is provided by informal caregivers [31, 32].

Under to the *Canada Health Act*, with the exception of physicians’ services, all services provided at the hospital without any cost to the patient are potentially subject to fees in ambulatory settings. Availability of and eligibility for publicly funded services varies across the 10 Canadian provinces and other national subsystems for nonhospital-based services. This situation generates a conflict when services are transferred from hospitals to homes and community settings.

Across provinces, regional organizations provide a single access point where applicants’ needs and eligibility criteria are assessed and matched to appropriate services, including home care, supportive living, or long-term care facilities [33]. Until April 2007, all provinces charged fees for HM services, while HHC services were provided with no charge. Consequently, there is an expectation that an income gradient in publicly funded HC receipt may be observed up to that date, but exclusively for HM services. After that, the governments of Ontario, Manitoba, Quebec, and PEI removed the co-pay requirement and currently do not charge any direct fees for HC services. The remaining six provinces, namely, British Columbia, Alberta, Saskatchewan, New Brunswick, Nova Scotia, and Newfoundland, have implemented income-testing procedures for the determination of HC fees. These testing procedures and corresponding differentiated fees may serve as disincentives to demand for services for higher income users and also remove barriers for low-income older adults.

## Conceptual framework

We used a household home care decision model previously developed by the authors [34], in which households allocate time and financial resources subject to resources and technology constraints.

The first component of the model that will be addressed in this study is the relationship between publicly funded HMPS and HHC services. Receipt of one is not expected to serve as a substitute for receipt of the other. Indeed, seniors receiving one kind of service are also expected to receive the other more intensively, given the fact that the main determinants of receipt of both HMPS and HHC services are similar and related mostly to age, dependence on help with ADLs/IADLs, and health status. However, when they are adjusted by these variables, they will not necessarily be complementary in their effects. This represents the first testable hypothesis in the model. If receipt of publicly funded HMPS services and receipt of publicly funded HHC services are complements, then receipt of one will be positively associated with receipt of the other, even after adjustments by functional and health status.

The second testable component comes from the model’s assumption that households fully exhaust their allocation of publicly funded HC services. The effect of receipt of informal care on publicly funded HMPS receipt, after adjustment for variables reflecting need, will be driven by the impact of this care on supply rather than on demand. Whether the dependent senior lives with other family members may influence his or her likelihood of receiving publicly funded HMPS services. For example, if help is needed for meal preparation, the possibility of being included in a “meals on wheels” program is higher if a person lives alone than if he or she lives with a functional partner. The probability of receiving help with household chores is also likely to be lower if the dependent senior is coresiding with children. Consequently, the availability of informal care from a coresident family member is expected to negatively affect the likelihood of receiving publicly funded HMPS services. A care receiver’s living arrangement will be closely related to the availability of help with ADLs/IADLs. Therefore, household arrangement may be considered a proxy for informal care from coresident caregivers and may be used to test this hypothesis. If informal HMPS services from coresident family members substitute for publicly funded HMPS services, living in a shared household arrangement will be negatively associated with the likelihood of receiving publicly funded HMPS services.

Another testable hypothesis is that allocation of publicly funded HC services is expected to be determined mostly by variables that reflect need (age, disability, dependence on help with ADLs, and chronic conditions). However, income is a constraint in the theoretical model, and so an effect of income on the likelihood of receiving publicly funded HMPS services is expected, due to scaled out-of-pocket contributions based on income assessments implemented in Canada over the study period.

## Methods

### Data and study population

Data for this study were derived from the household component of the National Population Health Survey (NPHS) held by Statistics Canada. The NPHS is a nationally representative longitudinal survey that collected data biennially from a panel of approximately 17,000 people for 18 years [35]. All nine waves of the NPHS were used for this study, covering the period from 1994-95 to 2010-11. The sample size ranged between 2302 and 2585 per wave, with a total of 7255 subjects included, and each one observed in 3 waves on average. A total of 22,490 observations were included in the analysis.

The study population was defined as people 65 years of age and older, who were residing in a community dwelling in one of the 10 Canadian provinces for at least one year during the study’s time frame. Individuals who turned 65 years of age during the course of the 16 years of observation were included for the waves in which they met the inclusion criteria. Individuals with incomplete follow-up information or who died were included in the waves in which data were available. This was therefore an unbalanced panel data set.

### Study variables

The outcome variables of the study were the probability of receipt of publicly funded HMPS, and the probability of receipt of HHC services. The NPHS also inquires about the type of services received and allows for more than one positive answer. The variables of the study are presented and described in Table 2. Household arrangement was used as a proxy for informal care.

**Table 2:** Study variables

Outcome variables	Variable type	Description
Receipt of publicly funded HHC services	Dichotomous	If there was a report of the receipt of nursing care and/or other health care services.
Receipt of publicly funded HMPS services	Dichotomous	If there was a report of the receipt of personal care, housework, meal preparation, or delivery, shopping, and/or respite care.
Explanatory Variables		
Household arrangement	Categorical: alone^a^, partner, or ‘other adult’	Living alone, living with a partner, or living with other adult but not with a partner. The cases when seniors were living with a partner and with other family members were included in the category “partner”. These categories were chosen on the basis of the conceptual model and according to frequency distribution.
Sex*partner	Interaction term	
Income	Categorical: low, middle^a^, or high	Categories were taken from the NPHS, which considers the household income adjusted for the number of household members. Grouping criteria were according to the frequency distribution of the survey variable.
Wave	Ordinal: 1 to 9	The “wave” variable included in each one of the adjusted panel data models was used to observe trends in the propensity to receive HC of each type over the study time frame and the trend’s statistical significance, adjusted by covariates.
Health status: diabetes, arthritis, heart disease, stroke, Alzheimer’s disease or other dementia, emphysema, cancer, and urinary incontinence.	Dichotomous	Health status was measured using dichotomous variables for several chronic conditions that may have important impacts on functional ability or that generate a need for health care services that may potentially be met at home (one variable per chronic condition).
More than 3 chronic conditions	Dichotomous	The presence of and interaction between multiple chronic conditions was considered through the inclusion of a binary variable if the individual indicated more than three chronic conditions.
Hospital	Dichotomous	If respondents had any overnight stays in a hospital in the last 12 months.
Disability	Dichotomous	If respondents had any long-term disabilities or handicaps.
Dependence	Categorical: high, middle, low, or no-dependence^a^	The NPHS measures need for help with five different ADLs:
		a) High-dependence: need for help with preparing meals or with personal care (such as washing, dressing, or eating) and/or moving around inside the house.
		b) Middle-dependence: no need for help with the previous two ADLs, but need for help with shopping for groceries or other necessities, and/or with doing normal, everyday housework.
		c) Low-dependence: no need for help with the previously mentioned four ADLs, but need for help with heavy household chores.
		d) No-dependence: no need for help with any of the above-mentioned ADLs.
Age	Continuous	In years.
Sex	Dichotomous	0 = female, 1 = male.
Minority	Dichotomous	Self identification as member of an ethnic minority.^b^
Immigrant	Dichotomous	Self identification as an immigrant.
Education	Dichotomous	0 = incomplete secondary education or lower; 1 = completed secondary education or higher.
Urban	Dichotomous	0 = rural; 1 = urban.
Province	Categorical: NF, PEI, NS, NB, QC, ON^a^, MA, SK, AL, or BC.	Province of residence at the point of inclusion in the sample.
Social support	Categorical: low, middle^a^, or high social support.	This variable captured elements of emotional and social support that are not essential elements of informal caregiving, but which may affect the likelihood of the receipt of HMPS services. This categorical variable was derived from a 16-category index.

^a^ Indicates reference category

^b^ Created according to the definition contained in the Canadian Employment Equity Act [47]

The inclusion of variables in the model was guided by the elements contained in the conceptual framework and grouping criteria were guided by frequency distribution. Alternative variable types and their different impacts on the model, and also interaction terms and collinearity were explored. For model selection criteria, an Akaike’s Information Criterion (AIC) was used.

### Statistical analysis

Simultaneous eligibility for HHC and HMPS services may possibly produce a problem of endogeneity when modelling the effect of one on the other, causing biased results due to the correlation of these predictors with the error term. Given these concerns, an instrumental variable (IV) approach was used.

Given the presence of binary outcome variables in this analysis, usual two-stage least square methods are unsuitable. Instead, a Two-Stage Residual Inclusion method was adopted. This approach has been used in health economics to address endogeneity issues through the use of IVs in nonlinear models [16, 22, 36, 37]. However, in the present analysis of a panel data set, the need to account for the time-invariant component of the error term made the use of these models unsuitable. Panel nonlinear regression models have the problem that they do not support two-stage methods, at least in the usual statistics packages, such as Stata. Using instrumental variable probit (ivprobit) in Stata, the problem of an invariant component of the error term across repeated observations remains unresolved, especially in this case, with nine waves of panel data. In addition, “ivprobit” requires the endogenous variable to be continuous, which was not the case in the current study.

### Panel two-stage residual inclusion

To address this problem, an original approach was used, referred to as the Panel Two-Stage Residual Inclusion model. We first specified a reduced form, first-step equation for each wave of the data set separately. Using logistic regression, we predicted the values of the endogenous variable, HMPS, as a function of its lagged values, and a set of explanatory variables:

$$ \boldsymbol{h}\boldsymbol{m}\boldsymbol{p}{\boldsymbol{s}}_{\boldsymbol{it}}=\boldsymbol{f}\ \left({\boldsymbol{\gamma}}_1 + {\boldsymbol{\gamma}}_2\boldsymbol{h}\boldsymbol{m}\boldsymbol{p}{\boldsymbol{s}}_{\boldsymbol{it}-2} + {\boldsymbol{\gamma}}_3\boldsymbol{alzheime}{\boldsymbol{r}}_{\boldsymbol{it}} + {\boldsymbol{\gamma}}_4\boldsymbol{social}\_\boldsymbol{l}\boldsymbol{o}{\boldsymbol{w}}_{\boldsymbol{it}} + {\boldsymbol{\gamma}}_4\boldsymbol{social}\_\boldsymbol{h}\boldsymbol{i}\boldsymbol{g}{\boldsymbol{h}}_{\boldsymbol{it}} + {\boldsymbol{\gamma}}_6{\boldsymbol{z}}_{\boldsymbol{it}}+{\boldsymbol{\varepsilon}}_{\boldsymbol{it}}\right) $$

*for t = waves {3, 4, 5, 6, 7, 8, 9} of the NPHS*^1^

where *HMPS* is receipt of publicly funded HMPS and *z* represents exogenous predictors (*age, sex, income, education, minority, immigrant, urban, partner, other adult, province, emphysema, diabetes, heart disease, stroke, incontinence, arthritis, disability, dependence, over 3 chronic conditions*). The IVs used exclusively for HMPS were second lagged values of HMPS receipt, dementia, and social support. From this regression, we obtained the residuals predicted for each wave $\left({\widehat{\boldsymbol{r}}}_{\boldsymbol{t}}^{\boldsymbol{hmps}}\right).$

Next we estimated an equation for likelihood of receipt of HHC, as a function of its second lagged values and other explanatory variables including whether the individual had cancer or had been hospitalized along with the same set of variables contained in z above:

$$ \boldsymbol{h}\boldsymbol{h}{\boldsymbol{c}}_{\boldsymbol{it}}=\boldsymbol{f}\ \left({\boldsymbol{\gamma}}_1+{\boldsymbol{\gamma}}_2\boldsymbol{h}\boldsymbol{h}{\boldsymbol{c}}_{\boldsymbol{it}-2} + {\boldsymbol{\gamma}}_3\boldsymbol{cance}{\boldsymbol{r}}_{\boldsymbol{it}} + {\boldsymbol{\gamma}}_4\boldsymbol{hospita}{\boldsymbol{l}}_{\boldsymbol{it}} + {\boldsymbol{\gamma}}_4{\boldsymbol{z}}_{\boldsymbol{it}}+{\boldsymbol{\varepsilon}}_{\boldsymbol{it}}\right) $$

*for t = waves {3, 4, 5, 6, 7, 8, 9} of the NPHS*

We generated the predicted residuals from this equation for each wave $\left({\widehat{\boldsymbol{r}}}_{\boldsymbol{t}}^{\boldsymbol{hhc}}\right).$ In both of the first stage equations we used second lagged values to avoid correlation with the error term in the second-stage equations.

#### Identification of endogenous predictors and exclusion restrictions

The following IVs were selected and exclusively used in one of the two first-step equations for the identification of each endogenous predictor separately:

- For *receipt of HHC*: second lagged values of HHC receipt, cancer, and hospital admission.

Receipt of publicly funded HHC services in previous cycles is expected to affect current HHC receipt yet not current HMPS. Regarding hospital admission, post-acute HC is prescribed after in-hospital stays to allow an early discharge through health services that can be delivered safely at home. Even though post-acute HMPS is also prescribed, these services are only provided concurrently with HHC after a hospital discharge. Canadian examples of these programs are *Aging at Home* and *Home First* in Ontario [38, 39]. Similarly, cancer patients managed at home receive professional services, and HMPS is only supplementary to HHC. In fact, in our sample, patients with an acute hospital admission in the past 12 months received 49 % more HHC than HMPS, a difference that reaches 71 % among cancer patients.

b) For *receipt of HMPS*: second lagged values of HMPS receipt, dementia, and social support.

Similarly, past HMPS receipt is expected to affect current HMPS receipt yet not the current receipt of HHC. In addition, home care services for dementia patients are mostly directed to provide support and respite to family caregivers [40] rather than HHC services. The variable *social support,* as previously described, captures elements of emotional and social support exclusively related to HMPS.

#### Second-stage equation

For the second-stage equations, panel logistic regression (“xtlogit” in Stata) with random-effects was used to specify receipt of HHC as a function of HMPS and other explanatory variables, and the residuals from the first stage HMPS equation described above were included to correct for the potential endogeneity of HMPS. We also specified HMPS as a function of HHC and other explanatory variables, including the residuals from the first stage HHC equation. Standard errors were estimated by bootstrapping. The second-stage HHC and HMPS equations were estimated as follows:

$$ \boldsymbol{h}\boldsymbol{h}{\boldsymbol{c}}_{\boldsymbol{it}}=\boldsymbol{f}\ \left({\boldsymbol{\beta}}_1 + {\boldsymbol{\beta}}_2\boldsymbol{h}\boldsymbol{m}\boldsymbol{p}{\boldsymbol{s}}_{\boldsymbol{it}} + {\boldsymbol{\beta}}_3{\boldsymbol{z}}_{\boldsymbol{it}}+{\boldsymbol{\beta}}_4\boldsymbol{cance}{\boldsymbol{r}}_{\boldsymbol{it}}+{\boldsymbol{\beta}}_5\boldsymbol{hospita}{\boldsymbol{l}}_{\boldsymbol{it}}+{\boldsymbol{\beta}}_6\boldsymbol{w}\boldsymbol{a}\boldsymbol{v}{\boldsymbol{e}}_{\boldsymbol{it}}+{\widehat{\boldsymbol{r}}}_{\boldsymbol{it}}^{\boldsymbol{hmps}}+{\boldsymbol{\varepsilon}}_{\boldsymbol{it}}\right) $$

$$ \boldsymbol{h}\boldsymbol{m}\boldsymbol{p}{\boldsymbol{s}}_{\boldsymbol{it}}=\boldsymbol{f}\ \left({\boldsymbol{\beta}}_1+{\boldsymbol{\beta}}_2\boldsymbol{h}\boldsymbol{h}{\boldsymbol{c}}_{\boldsymbol{it}}+{\boldsymbol{\beta}}_3\boldsymbol{alzheime}{\boldsymbol{r}}_{\boldsymbol{it}}+{\boldsymbol{\beta}}_4\boldsymbol{social}\_\boldsymbol{l}\boldsymbol{o}{\boldsymbol{w}}_{\boldsymbol{it}}+{\boldsymbol{\gamma}}_4\boldsymbol{social}\_{\boldsymbol{high}}_{\boldsymbol{it}}+{\boldsymbol{\beta}}_3{\boldsymbol{z}}_{\boldsymbol{it}}+{\boldsymbol{\beta}}_4\boldsymbol{w}\boldsymbol{a}\boldsymbol{v}{\boldsymbol{e}}_{\boldsymbol{it}}+{\widehat{\boldsymbol{r}}}_{\boldsymbol{it}}^{\boldsymbol{hhc}}+{\boldsymbol{\varepsilon}}_{\boldsymbol{it}}\right) $$

The significance of residual terms included in the second-stage equations was taken as a test for endogeneity [41]. To test the strength of the instrumental variables, nonlinearity prevented us from observing the F-test statistics. Instead, we tested the combined effect of the instrumental variables on the endogenous variable in the reduced form equations through chi-square tests. We also checked the increase in standard error, as compared with the noninstrumented model. A Hausman test was used to select random over fixed-effects.

To assess the robustness of the model the results were compared with an equivalent analysis using panel probit and Generalized Estimating Equations (GEE)^2^ specifications. In addition, the results were compared with those obtained from a noninstrumented panel logit approach. In general, for all the analyses performed, results were reported if a 10 % significance level was achieved.

All statistical analyses were performed using STATA 12.0 (StataCorp, 2011). Ethics approval for this research was granted by the Health Sciences Research Ethics Board of the University of Toronto (Protocol Reference # 27512, April 5, 2012).

## Results

Descriptive statistics are presented in Tables 3 and 4.^3^ The proportion of seniors in the sample who reported receipt of publicly funded HC services was 10.7 %, with 7.9 % receiving HMPS, 4.9 % HHC, and 2.1 % both. A total of 53.4 % lived with a partner; with the proportion of seniors living alone higher among those who received publicly funded HMPS services. An income gradient for HC receipt was evident in the comparative results.

**Table 3:** Descriptive statistics of the variables included in the adjusted analysis

Number of observations	22,490		
Number of subjects	7,255		
HC	10.7 %	Social support	
HHC	4.9 %	Low	4.7 %
HMPS	7.9 %	Middle	12.1 %
HHC & HMPS	2.1 %	High	83.2 %
Age	74.8 ± 7.2	Need help	40.4 %
Sex (male)	41.6 %	Moving inside the house	6.0 %
Urban	84.0 %	Preparing meals	9.3 %
Ethnic minority	15.4 %	Personal care	8.9 %
Immigrant status	24.4 %	Shopping	15.7 %
Living arrangement		Cleaning the house	17.0 %
Living alone	35.8 %	Heavy household duty	38.2 %
Living with partner	53.4 %	Dependence	
only with partner	47.1 %	No	59.6 %
with partner & other adult	6.3 %	Low	18.2 %
Living with other adult	10.8 %	Middle	10.7 %
Secondary education completed or higher	55.7 %	High	11.5 %
		Health status variables	
Income adequacy		Any chronic condition	87.7 %
Low	17.5 %	Number of chronic conditions	2.6 ± 2.0
Middle	37.7 %	0	12.7 %
High	44.9 %	1–3	59.6 %
Province		>3	27.7 %
NF	1.6 %	Hospitalization	11.8 %
PEI	0.4 %	Disability	29.6 %
NS	3.1 %	Diabetes	14.2 %
NB	2.6 %	Arthritis	46.9 %
QC	23.1 %	Heart Disease	18.0 %
ON	39.7 %	Stroke	5.0 %
MA	3.8 %	Dementia	3.6 %
SK	3.2 %	Emphysema	5.9 %
AL	8.5 %	Cancer	5.0 %
BC	14.0 %	Incontinence	10.8 %

**Table 4:** Comparative descriptive statistics by categories of HC receipt

		HMPS	No HMPS		HHC	No HHC	
Age		80.2 ± 0.17	73.9 ± 0.05	***	78.1 ± 0.23	74.2 ± 0.05	***
Sex (male)		32.9 %	42.9 %	***	40.6 %	42.2 %	
Minority		14.3 %	15.6 %		12.2 %	15.6 %	**
Immigrant		21.0 %	24.8 %	**	22.6 %	24.6 %	
Alone		54.3 %	31.7 %	***	43.9 %	32.9 %	***
Partner		36.0 %	57.1 %	***	43.5 %	56.0 %	***
Other_adult^a^		9.7 %	11.2 %		12.7 %	11.0 %	
Education^b^		50.0 %	56.2 %	***	47.6 %	56.1 %	***
Income^c^	Low	30.0 %	15.3 %	***	23.0 %	16.1 %	***
	Middle	43.2 %	37.3 %	***	47.3 %	37.3 %	***
	High	26.8 %	47.4 %	***	29.8 %	46.6 %	***
Urban		81.6 %	83.9 %	**	82.9 %	83.7 %	
Dependency	No	8.4 %	64.0 %	***	18.5 %	61.7 %	***
	Low	14.9 %	18.5 %	**	14.3 %	18.4 %	**
	Middle	25.6 %	9.4 %	***	19.7 %	10.3 %	***
	High	51.1 %	8.1 %	***	47.5 %	9.6 %	***
Over 3 chronic conditions		50.1 %	25.8 %	***	51.4 %	26.6 %	***
Disability		63.5 %	25.1 %	***	59.1 %	26.6 %	***
Diabetes		22.9 %	13.2 %	***	24.0 %	13.5 %	***
Heart disease		31.7 %	16.2 %	***	35.3 %	16.5 %	***
Stroke		13.6 %	3.6 %	***	13.4 %	3.9 %	***
Arthritis		64.0 %	45.0 %	***	61.2 %	45.7 %	***
Dementia		7.0 %	1.4 %	***	6.4 %	1.6 %	***
Emphysema		12.4 %	5.3 %	***	11.7 %	5.5 %	***
Cancer		8.0 %	4.7 %	***	13.7 %	4.5 %	***
Incontinence		19.4 %	8.3 %	***	20.8 %	8.5 %	***
Hospitalization		39.3 %	13.8 %	***	58.4 %	13.6 %	***
Social support	Low	10.4 %	5.5 %	***	6.8 %	5.8 %	
	Middle	17.1 %	12.6 %	**	15.1 %	12.8 %	
	High	72.5 %	81.9 %	***	78.2 %	81.4 %	

* Significant at the 10 % level. ** Significant at the 5 % level. *** Significant at the 1 % level

^a^ Living without a partner but with other adult(s)

^b^ Secondary education completed or higher

^c^ Household income adequacy (adjusted by household members)

In the panel logit two-stage residual inclusion analysis, the receipt of HMPS services significantly increased the likelihood of HHC receipt (OR = 3.85, *p* < 0.001; Table 5). Evidence of endogeneity of the variable HMPS was observed, with a significant t-statistic on the included residuals (*p* = 0.062), meaning that receipt of HHC was endogenous to receipt of HMPS services. The strength of the instruments was tested, and the average value of the chi-square test was 29.54 (*p* < 0.001; Table 6). In addition, the standard error of the correlation coefficient for the instrumented HMPS predictor was only 1.18 times larger than the noninstrumented variable. The instruments’ strength was considered adequate.

**Table 5:** HMPS and HHC receipt – second stage panel Logit analysis with and without IV

Dependent Variable →		HMPS				HHC			
		With IV		Without IV		With IV		Without IV	
		OR (SE)	*P*	OR (SE)	*p*	OR (SE)	*p*	OR (SE)	*p*
HMPS		--	--	--	--	3.848 (.982)	.000	4.850 (.566)	.000
HHC		13.71 (5.240)	.000	12.57 (2.509)	.000	--	--	--	--
Residuals HMPS		--	--	--	--	1.130 (.074)	.062	--	--
Residuals HHC		.944 (.103)	.595	--	--	--	--	--	--
Wave		.909 (.030)	.004	.912 (.030)	.005	.951 (.038)	.212	1.044 (.021)	.033
Income^a^	low	1.230 (.222)	.252	1.122 (.175)	.459	1.390 (.262)	.080	.969 (.117)	.795
	high	.541 (.099)	.001	.551 (.094)	.000	.635 (.107)	.007	.678 (.084)	.002
Education^b^		1.279 (.210)	.133	1.209 (.187)	.222	1.001 (.163)	.997	.968 (.103)	.763
Age		1.107 (.017)	.000	1.113 (.012)	.000	1.015 (.016)	.328	1.012 (.007)	.097
Sex (male)		1.872 (.428)	.006	2.050 (.417)	.000	1.550 (.314)	.030	1.234 (.184)	.158
Partner		.805 (.215)	.417	.642 (.138)	.039	.854 (.227)	.553	.831 (.133)	.246
Other_adult		.399 (.122)	.003	.340 (.112)	.001	1.309 (.357)	.323	1.285 (.225)	.153
Sex*partner		.232 (.085)	.000	.257 (.083)	.000	.820 (.306)	.591	1.147 (.250)	.529
Minority		.718 (.208)	.254	.753 (.197)	.278	.720 (.188)	.208	.932 (.160)	.681
Immigrant		.545 (.121)	.006	.514 (.115)	.003	.929 (.193)	.721	.844 (.121)	.237
Urban		.765 (.139)	.141	.728 (.133)	.082	.846 (.201)	.482	.911 (.110)	.436
Province^c^	NF	.212 (.106)	.002	.232 (.099)	.001	.201 (.114)	.005	.311 (.092)	.000
	PEI	.704 (.234)	.291	.716 (.226)	.290	.277 (.157)	.024	.405 (.098)	.000
	NS	.856 (.281)	.636	.825 (.251)	.527	.273 (.092)	.000	.284 (.070)	.000
	NB	.986 (.275)	.961	1.072 (.317)	.813	.299 (.116)	.002	.384 (.089)	.000
	QC	.428 (.112)	.001	.462 (.115)	.002	.970 (.240)	.903	1.123 (.172)	.449
	MA	.676 (.203)	.191	.726 (.219)	.288	.464 (.186)	.055	.543 (.112)	.003
	SK	.748 (.225)	.335	.676 (.197)	.180	.595 (.209)	.140	.697 (.134)	.060
	AL	.546 (.175)	.059	.514 (.163)	.036	.699 (.197)	.205	.637 (.133)	.031
	BC	.979 (.285)	.943	1.024 (.273)	.928	.282 (.109)	.001	.382 (.079)	.000
Dependence^d^	Low	3.870 (.640)	.000	4.010 (.726)	.000	2.061 (.425)	.000	1.894 (.276)	.000
	Middle	13.39 (2.817)	.000	13.36 (2.671)	.000	3.529 (.791)	.000	3.193 (.491)	.000
	High	36.39 (9.265)	.000	38.28 (8.337)	.000	4.410 (1.126)	.000	4.286 (.668)	.000
Disability		1.545 (.234)	.004	1.631 (.222)	.000	1.186 (.190)	.287	1.291 (.136)	.015
Over 3 chronic conditions		.910 (.157)	.584	.878 (.136)	.403	1.233 (.232)	.266	1.253 (.150)	.059
Diabetes		1.351 (.329)	.218	1.447 (.268)	.046	1.198 (.229)	.345	1.124 (.145)	.367
Heart disease		1.183 (.201)	.323	1.152 (.178)	.359	.987 (.201)	.949	1.112 (.124)	.340
Stroke		1.292 (.300)	.271	1.308 (.342)	.304	1.499 (.356)	.088	1.242 (.202)	.182
Arthritis		1.172 (.196)	.344	1.253 (.182)	.121	1.093 (.188)	.606	1.044 (.110)	.685
Emphysema		1.191 (.285)	.465	1.221 (.285)	.393	.701 (.176)	.158	.849 (.143)	.330
Incontinence		.908 (.186)	.637	.992 (.178)	.966	1.546 (.306)	.028	1.225 (.162)	.124
Dementia		.960 (.785)	.960	.931 (.592)	.911	--	--	--	--
Cancer		--	--	--	--	2.259 (.625)	.003	2.241 (.343)	.000
Hospitalization		--	--	--	--	7.377 (1.368)	.000	5.463 (.537)	.000
Social support	Low	.890 (.227)	.649	.803 (.194)	.364	--	--	--	--
	High	.837 (.144)	.302	.779 (.124)	.116	--	--	--	--

^a^ Reference category: middle-income adequacy

^b^ Secondary education completed or higher

^c^ Reference category: Ontario

^d^ Reference category: No dependency

**Table 6:** HMPS and HHC receipt – Chi-square results – First Stage Logit Analysis

Wave	HMPS IV	HHC IV
3	41.8*	50.5*
4	48.9*	46.6*
5	31.2*	40.0*
6	20.5*	46.0*
7	30.8*	62.7*
8	12.3*	31.0*
9	21.2*	53.2*
Average	29.5	47.1

*P-value <0.001

There was a positive and significant association between HHC receipt and the propensity to receive HMPS services (OR = 13.7, *p* < 0.001). However, the included residuals were nonsignificant at the 10 % level (*p* = 0.595). Given the simultaneous effect between receipt of HMPS and HHC services, and the significant evidence of endogeneity in the previous model, the instrumented model was preferred in both cases. Notwithstanding, the coefficients on the other variables in the model were, in general, stable when the residuals were included compared to when they were dropped out, as can be observed in Table 5. Regarding the strength of the instruments, the average value of the chi-square test was 47.13 (*p* < 0.001) and the standard error of the correlation coefficient for the instrumented HHC predictor was 0.91 times larger than the noninstrumented variable, which was considered adequate.

Household arrangement was a proxy for receipt of informal care in this analysis. In line with the hypothesis, living with other adult family members was associated with a significant decrease in the likelihood of receipt of publicly funded HMPS services (“other_adult”: OR = 0.40; *p* < 0.003). Living with a partner was found to be nonsignificant in general (“partner”: OR = 0.81; *p* = 0.417); however, the interaction term between sex (male) and living with partner was found to be significantly negative (“sex(male)*partner”: OR = 0.23; *p* < 0.001), a finding that may be interpreted as a substitution effect in relation to informal care provision only by a female partner for a male dependent. As expected on the basis of the conceptual framework, the availability of informal care was nonsignificant in affecting HHC receipt.

The income variable had a significant effect on the propensity to receive HMPS services, with significantly fewer services for higher-income people (OR = 0.54, *p* = 0.001), as compared with the middle income-adequacy category, and with no observed significant difference between low- and middle-income seniors. Unexpectedly, high-income seniors reported a significantly lower likelihood of HHC receipt (OR = 0.64, *p* = 0.007), and low-income seniors reported a significantly higher likelihood of HHC receipt (OR = 1.39, *p* = 0.080).

Age, disability, and dependency were strong predictors of publicly funded HMPS receipt, while stroke, incontinence, cancer, dependency and hospitalization were associated with a higher likelihood of HHC receipt. The analysis showed an increased likelihood of HMPS and HHC receipt by male seniors. Immigrant status was found to be associated with a significantly lower likelihood of HMPS receipt, in spite of the fact that the average time since immigration in our sample was 43.9 years. In contrast, ethnic minority was not a significant predictor in any of the analyses.

Among other demographic variables, urban seniors did not report a significantly different likelihood of the receipt of HC services. All provinces showed a lower likelihood of HC receipt as compared to Ontario. However, the sample sizes were limited for the small provinces. Among the four provinces that had higher representation in the sample, residents of Quebec and Alberta had a significantly lower likelihood of HMPS receipt in the adjusted analysis, and residents of British Columbia had a significantly lower likelihood of HHC receipt, as compared to Ontario residents.

The proportion of seniors reporting receipt of public HMPS services decreased significantly over the study’s time frame in the adjusted analysis. Receipt of HHC, in contrast, did not show a significant variation.

Even though the trends in the association between predictors and HC receipt did not change after the inclusion of residuals, there were some changes in the significance of variables, as shown in the second and fourth columns of Table 5. Results from the first stage logit equations are included as Appendices.

The results from the preferred model were entirely comparable in terms of the magnitude and direction of effects with those obtained using a panel probit model and weighted GEE analysis in the second-stage equation, although significance levels showed some differences. These results are available upon request.

## Discussion

The HC literature, up to this point, most frequently treats HHC and HMPS as one homogeneous type of service. Findings in our study support the existence of important differences among them. This research provides robust empirical evidence to confirm part of the hypotheses in our conceptual framework, which translates into a number of important policy implications. In addition, this study is the first attempt to explore and model the potential system of interactions between HC services of different types.

We found that receipt of publicly funded HHC was complementary to the receipt of publicly funded HMPS services. This finding supports the assumption that, once a senior accesses the HC system, the probability of being deemed eligible or being offered additional services is higher. The receipt of HMPS was also a strong predictor of HHC receipt, although weaker than the effect of HHC as a predictor of HMPS receipt. This finding partially departed from the expectation that receipt of HMPS would be a weak predictor of HHC receipt. A certain level of qualification is required for assessment of the need for health services, and this type of care is specialized, as compared with HMPS services.

In terms of patient care, the consequence of this complementary effect is an increased gap between care recipients and non-recipients, who are at equivalent levels of functional and health status. This element raises concerns about equitable access to HC services in Canada, especially given that provincial HC programs are characterized by capped budgets and overall service volume constraints [8]. This finding also raises concerns worth exploring in other systems. In Europe and among US Medicare beneficiaries, this complementary effect may be increasing inequitable access to services, an element not yet studied in these jurisdictions.

Consistent with the theoretical model, we found that the availability of informal care was a negative and significant determinant of the receipt of publicly funded HMPS services, when such care was included in the analysis through the use of household arrangement variables. However, living with a partner only significantly reduced the likelihood of receipt of HMPS when the caregiver partner was a female. Living with other adult family members significantly reduced the likelihood of receipt of HMPS. These findings confirm the reliance on family caregiving when allocating publicly funded HC services, even if eligibility criteria are not explicit in this regard; gender differences are also confirmed. Living arrangement variables were not significant predictors of HHC receipt, which represent additional evidence to the importance of analyzing different types of HC services separately. These observations were concurrent with the interactions in the conceptual framework. Observed differences in the determinants of the receipt of HHC and HMPS may translate into unattended needs in systems where eligibility to HC services is bundled for health and social care, such as the US Medicare system –a hypothesis worth exploring further.

Income was an important determinant of HMPS receipt, with fewer services for high-income seniors, as compared with those in the middle- and low-income categories. These findings were expected because all Canadian provinces charged copayments for HMPS services until 2007, payments that were graduated by income [33]. In addition, the probability of the substitution by privately paid HMPS was also hypothesized to be higher for higher-income seniors. Unexpectedly, we also observed differences according to income in the receipt of HHC services even after we included a large set of controls for health status, with wealthier individuals less likely to receive services. This is somewhat surprising since these services are provided free of charge in every province, regardless of the ability to pay.

The study has several limitations, mostly related to the data source. First, the NPHS asks if subjects received services at home or not, but does not inquire about the intensity of services received, i.e., total amount of hours of care received. Second, receipt of informal care from a coresident family member was only inferred from its availability in terms of living arrangement, since specific information on the actual receipt of informal care was not available at this point. In addition, the NPHS has a reduced number of questions specific to HC receipt, which limited the scope of questions to be addressed with this data set.

Among the contributions, this study uses 18 years of longitudinal data, which represent an extraordinary opportunity to explore the determinants and interactions in the receipt of HC services. The results suggest that there are important differences between the drivers of HHC and HMPS service receipt. As regards, the second aim—to explore the potential interactions between the likelihood of receipt of the two types of services, this study represents a first attempt to model the determinants of types of publicly funded HC receipt as a system. We argued that failure to address the potential for interactive effects in the existing literature may have biased findings to date. The challenge in this study was that the set of potential instruments in the data were few and necessitated use of lagged variables. We explored various specifications to test the robustness of our results and tested the instruments for strength and validity.

Our results remained consistent across the instrumented and noninstrumented approaches, confirming our main research hypotheses. Therefore, the policy implications discussed deserve careful consideration from health care providers and policy makers, to ensure equitable and fair access to health and social support in the home and community settings for the frail elders. Nevertheless, future research should further explore the questions posed within the framework presented in this paper using data that offer a wider set of potentially strong instruments.

The original methodological approach proposed in this study, a Panel Two-Stage Residual Inclusion approach, seems to have yielded sensible results and to have effectively addressed problems that are known to be a source of bias in the literature.

## Conclusions

A conclusion of this study is that the distinction between receipt of HHC and HMPS services matters, given the fact that we found differences in the determinants of the likelihood of HHC and HMPS receipt.

We found a complementary interrelationship between receipt of publicly funded HMPS and HHC services, after adjusting for functional and health status. The consequence of this complementary effect may be an increased gap between care recipients and non-recipients, who are at equivalent levels of functional and health status.

Dependence on help with activities of daily living, health status, household arrangement, and income were significant determinants of the propensity to receive both publicly funded HHC and HMPS services.

Our results suggest that the determinants of receipt of HC services of different types are more complex than has been acknowledge so far, and that this complexity should be taken into greater consideration in the empirical literature.

## Appendices

**Table 7:** First stage Logit equations –HHC receipt (waves 3 to 6)

Dependent Variable →		HHC				HHC			
		Wave 3		Wave 4		Wave 5		Wave 6	
		OR (SE)	*P*	OR (SE)	*p*	OR (SE)	*p*	OR (SE)	*P*
HHC _t-2_		1.945 (.987)	.189	3.309 (1.575)	.012	2.289 (1.068)	.076	6.873 (3.131)	.000
Cancer		.841 (.361)	.687	.774 (.363)	.599	2.434 (1.151)	.069	2.200 (.966)	.072
Hospitalization		5.328 (1.267)	.000	5.606 (1.457)	.000	5.630 (1.701)	.000	5.771 (1.755)	.000
Income^a^	Low	1.248 (.332)	.404	.606 (.188)	.107	2.220 (.789)	.025	1.689 (.627)	.158
	High	.481 (.173)	.042	.714 (.227)	.289	.743 (.277)	.426	.600 (.229)	.180
Education^b^		.939 (.224)	.792	.726 (.193)	.228	1.067 (.321)	.829	1.054 (.317)	.861
Age		1.014 (.020)	.462	1.046 (.022)	.030	1.067 (.025)	.005	1.038 (.025)	.120
Sex (male)		.875 (.313)	.710	2.706 (.914)	.003	1.576 (.629)	.255	1.254 (.531)	.593
Partner		.754 (.280)	.448	.746 (.318)	.492	1.239 (.607)	.661	1.663 (.814)	.299
Other_adult		.366 (.205)	.073	.930 (.336)	.646	3.296 (1.648)	.017	1.589 (.793)	.354
Sex*partner		1.280 (.685)	.645	.294 (.172)	.036	.943 (.605)	.928	.688 (.448)	.565
Minority		1.013 (.422)	.974	.981 (.469)	.968	.401 (.230)	.111	.881 (.460)	.808
Immigrant		.754 (.252)	.400	.380 (.151)	.015	.755 (.337)	.529	1.355 (.518)	.426
Urban		.784 (.239)	.424	1.011 (.344)	.974	1.019 (.378)	.960	.732 (.296)	.440
Province^c^	NF	.462 (.281)	.205	.068 (.078)	.019	.299 (.283)	.202	.154 (.169)	.088
	PEI	.146 (.115)	.015	.382 (.201)	.067	.321 (.233)	.117	.922 (.543)	.890
	NS	.586 (.270)	.246	.337 (.188)	.052	.111 (.117)	.038	.101 (.111)	.036
	NB	.187 (.112)	.005	.786 (.380)	.618	.574 (.415)	.443	.182 (.161)	.053
	QC	1.550 (.524)	.195	.504 (.194)	.075	1.604 (.660)	.250	1.430 (.604)	.398
	MA	.448 (.196)	.066	.369 (.198)	.064	.469 (.290)	.221	.530 (.366)	.358
	SK	.937 (.386)	.874	.356 (.179)	.040	1.760 (.816)	.223	.437 (.277)	.191
	AL	.337 (.195)	.060	.320 (.183)	.046	1.428 (.787)	.518	.678 (.349)	.450
	BC	.438 (.208)	.082	.285 (.156)	.022	.493 (.303)	.249	.420 (.257)	.156
Dependence^d^	Low	3.857 (1.525)	.001	1.473 (.587)	.331	2.413 (1.145)	.063	.980 (.526)	.970
	Middle	7.068 (2.906)	.000	3.474 (1.373)	.002	3.882 (1.915)	.006	4.611 (2.167)	.001
	High	15.02 (6.109)	.000	4.024 (1.616)	.001	4.727 (2.284)	.001	4.019 (1.960)	.004
Disability		.935 (.246)	.800	1.820 (.513)	.034	1.406 (.438)	.273	1.352 (.431)	.344
Over 3 chronic conditions		1.303 (.370)	.351	1.792 (.582)	.072	.910 (.338)	.800	1.531 (.557)	.242
Diabetes		1.588 (.468)	.116	1.064 (.344)	.848	1.648 (.562)	.143	1.013 (.357)	.970
Heart disease		.918 (.245)	.750	.824 (.247)	.518	.778 (.257)	.448	1.129 (.385)	.721
Stroke		1.127 (.418)	.748	1.572 (.607)	.242	1.122 (.521)	.805	1.731 (.741)	.200
Arthritis		1.361 (.346)	.225	.774 (.210)	.346	.967 (.303)	.914	.989 (.316)	.973
Emphysema		1.811 (.327)	.603	1.369 (.555)	.438	.294 (.169)	.033	.399 (.233)	.115
Incontinence		2.868 (.847)	.000	.715 (.296)	.418	2.351 (.834)	.016	.479 (.201)	.080

^a^ Reference category: middle-income adequacy

^b^ Secondary education completed or higher

^c^ Reference category: Ontario

^d^ Reference category: No dependency

**Table 8:** First stage Logit equations –HHC receipt (waves 7 to 9)

Dependent Variable →		HHC					
		Wave 7		Wave 8		Wave 9	
		OR (SE)	*P*	OR (SE)	*p*	OR (SE)	*p*
HHC _t-2_		1.063 (.765)	.932	2.416 (1.441)	.139	5.413 (3.107)	.003
Cancer		1.307 (.661)	.597	4.535 (2.342)	.003	5.963 (2.672)	.000
Hospitalization		10.74 (3.273)	.000	5.469 (1.983)	.000	8.178 (2.883)	.000
Income^a^	Low	.848 (.359)	.698	1.032 (.565)	.953	1.184 (.607)	.742
	High	.626 (.214)	.172	.875 (.355)	.742	.380 (.155)	.018
Education^b^		1.244 (.381)	.476	1.395 (.507)	.360	1.614 (.584)	.185
Age		1.005 (.025)	.858	.987 (.026)	.627	.978 (.027)	.413
Sex (male)		.976 (.477)	.960	1.194 (.655)	.746	.875 (.504)	.817
Partner		.735 (.355)	.523	.336 (.190)	.054	.670 (.368)	.465
Other_adult		.611 (.345)	.383	.298 (.212)	.088	1.507 (.973)	.525
Sex*partner		1.643 (1.094)	.456	1.095 (.873)	.909	2.519 (2.008)	.247
Minority		1.020 (.480)	.966	.904 (.445)	.837	.278 (.186)	.055
Immigrant		.733 (.325)	.483	2.135 (.909)	.075	1.272 (.572)	.593
Urban		.609 (.243)	.213	.692 (.338)	.451	1.150 (.607)	.791
Province^c^	NF	.233 (.251)	.177	1 (omitted)		.732 (.608)	.707
	PEI	.296 (.218)	.099	1 (omitted)		.638 (.576)	.619
	NS	.285 (.188)	.057	.316 (.257)	.156	.292 (.324)	.267
	NB	.444 (.278)	.194	.696 (.448)	.573	1.595 (1.052)	.479
	QC	1.961 (.839)	.115	.444 (.248)	.146	1.415 (.742)	.508
	MA	.825 (.502)	.752	.899 (.536)	.858	1.278 (.796)	.694
	SK	.769 (.436)	.643	.726 (.446)	.603	.891 (.702)	.883
	AL	.713 (.420)	.565	.299 (.225)	.109	1.946 (1.143)	.261
	BC	.542 (.336)	.324	.431 (.308)	.239	.319 (.276)	.187
Dependence^d^	Low	1.637 (.719)	.262	3.044 (1.603)	.035	2.065 (1.213)	.217
	Middle	2.017 (.953)	.138	3.625 (2.097)	.026	6.171 (3.685)	.002
	High	7.913 (3.511)	.000	6.633 (4.005)	.002	9.500 (5.707)	.000
Disability		1.181 (.373)	.599	1.943 (.734)	.079	1.393 (.562)	.411
Over 3 chronic conditions		1.122 (.416)	.756	.810 (.371)	.645	.660 (.279)	.325
Diabetes		1.246 (.457)	.549	1.142 (.497)	.761	.757 (.338)	.532
Heart disease		.842 (.297)	.625	2.799 (1.106)	.009	1.232 (.467)	.582
Stroke		.432 (.269)	.178	2.202 (1.134)	.125	1.896 (1.027)	.238
Arthritis		.954 (.319)	.889	1.137 (.437)	.739	1.407 (.553)	.385
Emphysema		1.110 (.596)	.847	1.262 (.789)	.709	.608 (.399)	.448
Incontinence		.791 (.321)	.563	1.168 (.482)	.706	1.155 (.464)	.721

^a^ Reference category: middle-income adequacy

^b^ Secondary education completed or higher

^c^ Reference category: Ontario

^d^ Reference category: No dependency

**Table 9:** First stage Logit equations –HMPS receipt (waves 3 to 6)

Dependent Variable →		HMPS				HMPS			
		Wave 3		Wave 4		Wave 5		Wave 6	
		OR (SE)	*P*	OR (SE)	*p*	OR (SE)	*p*	OR (SE)	*P*
HMPS _t-2_		4.704 (1.319)	.000	8.883 (2.812)	.000	6.138 (2.018)	.000	4.580 (1.648)	.000
Alzheimer		3.057 (2.366)	.149	1 (omitted)		1 (omitted)		.977 (1.228)	.985
Social support	Low	.573 (.214)	.136	1.151 (.550)	.768	1.552 (.746)	.360	2.684 (1.345)	.049
	High	.438 (109)	.001	1.535 (.509)	.196	.1.319 (447)	.414	1.202 (.457)	.629
Income^a^	Low	.816 (.194)	.392	1.070 (.274)	.792	1.827 (.568)	.052	1.696 (.534)	.094
	High	.493 (.148)	.018	.529 (.163)	.039	.661 (.229)	.232	.533 (.197)	.089
Education^b^		1.382 (.294)	.128	1.061 (.249)	.799	1.326 (.367)	.308	1.176 (.314)	.543
Age		1.040 (.017)	.017	1.051 (.020)	.010	1.074 (.023)	.001	1.062 (.024)	.007
Sex (male)		1.325 (.393)	.343	1.333 (.420)	.362	2.359 (.832)	.015	2.749 (.991)	.005
Partner		.857 (.270)	.624	.658 (.227)	.225	.939 (.384)	.879	.921 (.405)	.852
Other_adult		.336 (.169)	.030	.582 (.256)	.219	.457 (.305)	.241	1.525 (.737)	.382
Sex*partner		.511 (.249)	.169	.279 (.159)	.025	.310 (.193)	.060	.286 (.186)	.054
Minority		.601 (.231)	.186	.502 (.227)	.128	1.381 (.593)	.452	1.701 (.713)	.205
Immigrant		.873 (.245)	.629	.528 (.185)	.068	.363 (.154)	.017	.798 (.316)	.570
Urban		.762 (.201)	.301	.660 (.193)	.156	.802 (.276)	.521	1.352 (.531)	.442
Province^c^	NF	.114 (.093)	.007	.071 (.062)	.003	.629 (.471)	.536	.677 (.447)	.554
	PEI	.706 (.294)	.403	.493 (.220)	.113	.552 (.344)	.340	1.247 (.663)	.678
	NS	1.120 (.440)	.773	.716 (.306)	.435	.826 (.428)	.712	.615 (.347)	.389
	NB	1.027 (.395)	.944	.495 (.243)	.152	1.074 (.555)	.890	1.471 (.739)	.442
	QC	.417 (.169)	.031	.411 (.157)	.020	.599 (.257)	.232	.613 (.275)	.275
	MA	.494 (.196)	.031	.949 (.446)	.911	.646 (.402)	.483	.467 (.291)	.222
	SK	1.477 (.515)	.263	.549 (.243)	.176	1.655 (.761)	.273	.405 (.254)	.150
	AL	.620 (.273)	.278	.897 (.422)	.818	1.574 (.773)	.356	.626 (.335)	.381
	BC	.815 (.301)	.580	1.122 (.427)	.761	1.516 (.683)	.355	.635 (.292)	.324
Dependence^d^	Low	4.577 (1.536)	.000	2.747 (.868)	.001	5.453 (2.549)	.000	3.937 (.1.806)	.003
	Middle	12.30 (4.268)	.000	6.923 (2.403)	.000	25.32 (11.84)	.000	10.04 (4.647)	.000
	High	45.35 (16.36)	.000	15.66 (5.619)	.000	31.73 (15.67)	.000	24.09 (11.55)	.000
Disability		1.077 (.242)	.740	1.660 (.423)	.047	1.136 (.314)	.646	1.350 (.379)	.286
Over 3 chronic conditions		1.242 (.303)	.374	1.353 (.384)	.287	.680 (.216)	.226	1.141 (.373)	.687
Diabetes		1.593 (.454)	.102	1.289 (.383)	.394	.819 (.281)	.560	1.245 (.402)	.498
Heart disease		1.273 (.301)	.307	.942 (.254)	.825	1.340 (.377)	.299	1.205 (.370)	.544
Stroke		1.305 (.453)	.443	.877 (.349)	.742	1.473 (.657)	.386	.965 (.489)	.944
Arthritis		.990 (.221)	.963	.756 (.185)	.252	1.094 (.316)	.755	.931 (.281)	.811
Emphysema		.640 (.241)	.236	.828 (.358)	.663	1.666 (.743)	.253	1.678 (.728)	.233
Incontinence		.940 (.307)	.849	.959 (.346)	.907	.900 (.330)	.774	.756 (.275)	.442

^a^ Reference category: middle-income adequacy

^b^ Secondary education completed or higher

^c^ Reference category: Ontario

^d^ Reference category: No dependency

**Table 10:** First stage Logit equations –HMPS receipt (waves 7 to 9)

Dependent Variable →		HMPS					
		Wave 7		Wave 8		Wave 9	
		OR (SE)	*P*	OR (SE)	*p*	OR (SE)	*p*
HMPS _t-2_		15.75 (7.871)	.000	6.667 (4.068)	.002	10.51 (5.612)	.000
Alzheimer		4.780 (8.122)	.357	1 (omitted)		1.087 (1.193)	.940
Social support	Low	2.390 (1.749)	.234	.491 (.396)	.377	.298 (.252)	.152
	High	1.993 (1.003)	.170	1.599 (.718)	.296	1.067 (.459)	879
Income^a^	Low	1.084 (.503)	.862	1.638 (.779)	.299	1.415 (.714)	.492
	High	.784 (.345)	.580	1.204 (.448)	.617	.728 (.291)	.428
Education^b^		.467 (.170)	.037	.688 (.225)	.253	1.176 (.426)	.654
Age		1.088 (.033)	.006	1.096 (.029)	.001	1.138 (.035)	.000
Sex (male)		2.009 (1.021)	.170	1.897 (.960)	.206	5.662 (2.723)	.000
Partner		.716 (.418)	.568	.815 (.376)	.657	.977 (.525)	.966
Other_adult		.894 (.653)	.878	.281 (.215)	.097	.140 (.158)	.081
Sex*partner		.602 (.477)	.522	.308 (.226)	.108	.087 (.070)	.002
Minority		1.088 (.588)	.877	.760 (.438)	.634	.529 (.355)	.343
Immigrant		.955 (.485)	.927	.976 (.452)	.958	.411 (.277)	.188
Urban		1.175 (.626)	.763	.648 (.283)	.321	.640 (.314)	.363
Province^c^	NF	.600 (.523)	.558	.325 (.282)	.194	1.522 (1.360)	.639
	PEI	.702 (.444)	.576	1.118 (.725)	.864	2.134 (1.646)	.326
	NS	.521 (.370)	.359	.649 (.410)	.494	2.283 (1.580)	.233
	NB	.633 (.385)	.452	.792 (.531)	.727	4.714 (3.101)	.018
	QC	.462 (.276)	.196	1.301 (.626)	.584	1.902 (1.099)	.266
	MA	.314 (.290)	.210	.487 (.322)	.276	7.091 (4.689)	.003
	SK	.211 (.171)	.055	.557 (.379)	.390	.808 (.720)	.811
	AL	.199 (.173)	.063	.563 (.399)	.418	1.976 (1.590)	.397
	BC	.541 (.354)	.348	.795 (.528)	.730	1.753 (1.216)	.419
Dependence^d^	Low	2.346 (1.268)	.114	5.815 (2.814)	.000	1.453 (.755)	.473
	Middle	6.716 (3.379)	.000	13.71 (7.038)	.000	7.019 (3.788)	.000
	High	16.56 (9.262)	.000	54.91 (34.15)	.000	9.383 (5.315)	.000
Disability		1.579 (.581)	.214	1.145 (.382)	.685	1.107 (.425)	.791
Over 3 chronic conditions		.714 (.302)	.426	.613 (.245)	.221	.554 (.244)	.180
Diabetes		.880 (.421)	.790	1.294 (.539)	.536	3.492 (1.549)	.005
Heart disease		.609 (.264)	.252	.915 (.362)	.823	1.538 (.612)	.279
Stroke		.381 (.352)	.297	.192 (.172)	.066	1.041 (.704)	.953
Arthritis		2.272 (.962)	.053	1.602 (.571)	.187	2.136 (.853)	.057
Emphysema		.437 (.357)	.311	1.508 (.845)	.463	1.020 (.712)	.978
Incontinence		.945 (.456)	.907	.380 (.183)	.044	2.254 (.960)	.056

^a^ Reference category: middle-income adequacy

^b^ Secondary education completed or higher

^c^ Reference category: Ontario

^d^ Reference category: No dependency

## Abbreviations

ADLs/IADLs: activities of daily living/instrumental activities of daily living
AHEAD: asset and health dynamics among the oldest-old
AIC: Akaike’s information criterion
AL: Alberta
BC: British Columbia
GEE: generalized estimating equations
HC: home care
HHC: home health care
HMPS: homemaking/personal support
IV: instrumental variable
MA: Manitoba
NB: New Brunswick
NF: Newfoundland & Labrador
NPHS: national population health survey
NS: Nova Scotia
ON: Ontario
PEI: Prince Edward Island
QC: Québec
SHARE: survey of health, ageing and retirement in Europe
SK: Saskatchewan

## Footnotes

1 The use of second lagged values in the first-stage equations requires missing waves 1 and 2 in the instrumented model.

2 The survey weights used for the sampling strategy were included in the GEE model.

3 Descriptive results are presented using the total number of observations as the denominator (as opposed to total number of individuals), and after adjusting by survey weights.
